# Multiple Orthokeratinized Odontogenic Cysts in a Female Adolescent Patient: A Report of a Rare Case

**DOI:** 10.7759/cureus.99771

**Published:** 2025-12-21

**Authors:** Kanimozhiy Senguttuvan, Senthil Kumar, Satish Kumar

**Affiliations:** 1 Oral and Maxillofacial Surgery, Thai Moogambigai Dental College and Hospital, Chennai, IND

**Keywords:** cyst enucleation, mandibular jaw cysts, odontogenic cyst, odontogenic keratocyst, orthokeratinized odontogenic cyst

## Abstract

An orthokeratinized odontogenic cyst (OOC) is a rare developmental odontogenic cyst characterized by orthokeratinized stratified squamous epithelium. It was previously considered a variant of an odontogenic keratocyst (OKC) but has since been classified as a separate entity due to differing biological behavior. We present a case of an OOC in a 17-year-old female patient with extra-oral swelling on her right ramus region. Radiolucency in both the jaws was discovered incidentally on routine OPG examination. These lesions were surgically enucleated, and histopathological examination confirmed the diagnosis. This report highlights the clinical, radiographic, and histological characteristics of OOCs with their rarity of multiple incidence and reviews pertinent literature.

## Introduction

An orthokeratinized odontogenic cyst (OOC) is a rare developmental cyst of odontogenic origin characterized by an orthokeratinized epithelial lining, which is biologically less aggressive than an odontogenic keratocyst (OKC). Initially described by Wright in 1981, it was formerly considered a subtype of the OKC but is now classified as a distinct entity by the WHO in 2017. OOCs exhibit distinctive and unique clinical, histopathological features and biological behavior that vary from OKCs. Differentiating OOCs from OKCs is clinically significant because the treatment approach, follow-up protocol, and recurrence risk differ substantially [1].

OKCs overall account for approximately 11% of all odontogenic cysts. OOCs are much less common than OKCs, constituting less than 1% of all odontogenic cysts and 0.4-10% of OKCs. They tend to occur in the posterior mandible in almost 90% of cases, often in association with impacted teeth, and maxillary involvement is uncommon (only ~9% of cases reported) [2]. They typically occur in adults in the 3rd -4th decades (mean age ~33 39 years), though variations exist. The overall epidemiological pattern underscores their rarity and benign clinical course compared with other jaw cysts [3]. These cysts are usually asymptomatic and discovered incidentally on radiographic examination. Differentiation from other cysts, particularly OKCs and dentigerous cysts, is essential due to differing recurrence rates (much lower than OKCs, which range from 12 to 60%) and less aggressive treatment protocols compared to OKCs [4].

As multiple OOCs are rare, this case report highlights an unusual presentation and underscores the importance of accurate diagnosis.

## Case presentation

Patient information

A 17-year-old girl with no relevant medical history presented to our dental hospital with a 4cm x 3cm extraoral swelling present in the right ramus region of the mandible extending from the tragus of the ear to the lower border of the mandible for the past one week. It was non-pulsatile, and the skin over the swelling was smooth. Mouth opening was restricted.

On palpation, the swelling was firm in consistency, warm to the touch, and the overlying skin was pinchable. She reported no pain or other associated symptoms.

Clinical and radiographic findings

**Figure 1:**
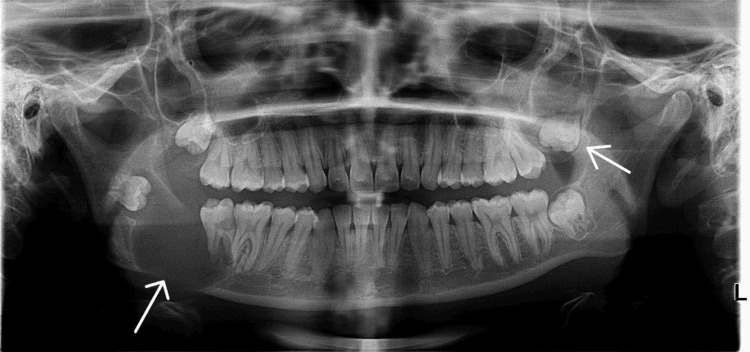
OPG The orthopantomogram (OPG) reveals a well-defined unilocular radiolucent lesion associated with the impacted tooth in the mandible with cortical expansion and well-defined radiolucency surrounding the upper left third molar.

Intraoral examination was slightly remarkable with vestibular obliteration with mild mucosal stretching. A panoramic radiograph revealed a well-defined unilocular radiolucency measuring approximately 3.5cm × 3 cm in the right posterior mandible, associated with an impacted right mandibular third molar and a radiolucency in the left maxilla, approximately measuring 1.5cm x 1 cm associated with an impacted left maxillary third molar (Figure 1). No root resorption or perforation was noted. Though perforation was not noted, there was significant thinning of the lower border of the mandible, which is a significant finding. Also, the third molar is displaced, which states the aggressive behavior of the lesion.

**Figure 2:**
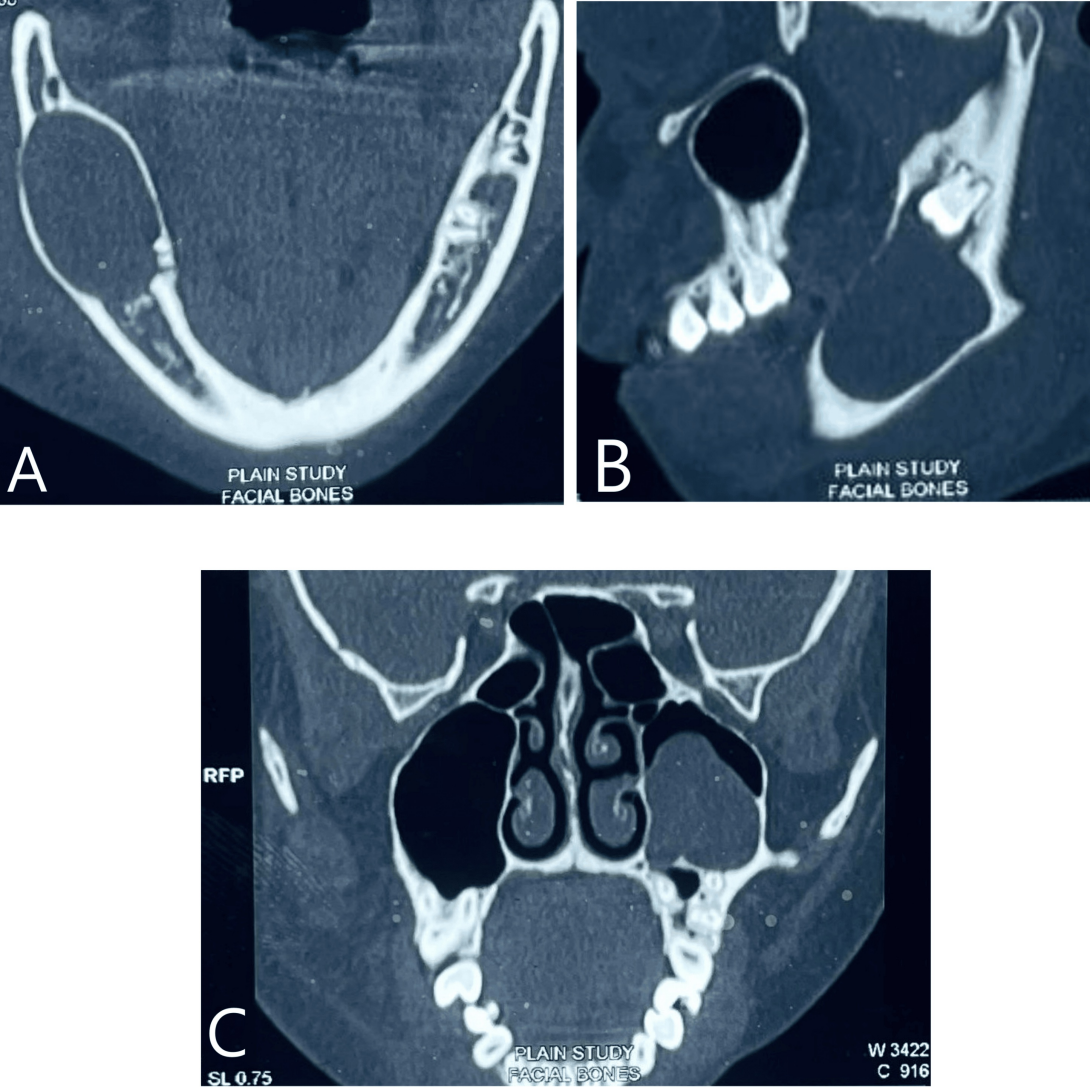
CT revealing radiolucent cystic lesions in both the maxilla and mandible A. Axial CT of the mandible reveals bicortical bone expansion due to the presence of a cystic lesion. B. Sagittal-section CT of the mandible reveals a large hypodensity extending along the ramus of the mandible associated with the impacted third molar. C. Coronal-section CT shows a hypodense mass associated with the maxillary third molar.

CT revealed hypodensity in the right mandibular ramus region with cortical expansion and a radiolucent lesion in the left maxillary region associated with impacted 28 (Figure 2).

Provisional diagnosis

A dentigerous cyst was considered based on clinical and radiographic findings.

Therapeutic intervention

In the right mandible, a trapezoidal flap was elevated, and a window was created to access the cystic lining and complete enucleation of the cystic wall was done. Carefully, 48 was located and extracted. Extraction of 47 and 46 was also done because of cystic involvement. Chemical cauterization was done using modified Carnoy’s solution. Platelet-rich fibrin (PRF) was placed, and wound closure was done using absorbable sutures.

**Figure 3:**
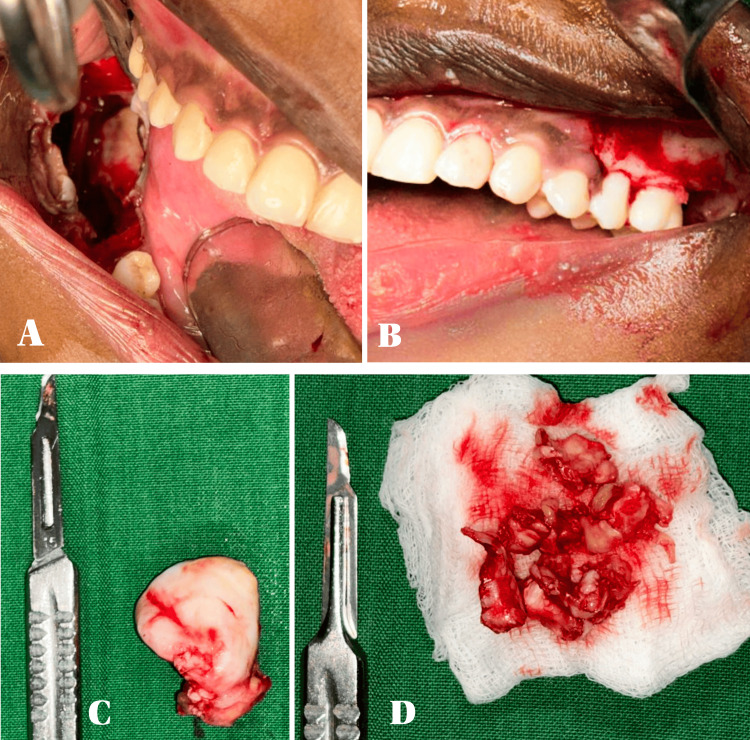
Intra-operative pictures A. Enucleation of the cyst and chemical cauterization done with Carnoy's solution in the right ramus of the mandible. B. Figure showing a cyst enucleated cavity after Carnoy's solution in the upper left third molar region. C. Maxillary enucleated cystic specimen. D. Mandibular cystic specimen along with involved teeth.

In the left maxilla, a triangular flap extending from the second premolar up to the tuberosity was raised, and the impacted tooth, along with the cystic lining, was identified and enucleated. Chemical cauterization and PRF placement with wound closure were done as already explained above (Figure 3).

Histopathological findings

**Figure 4:**
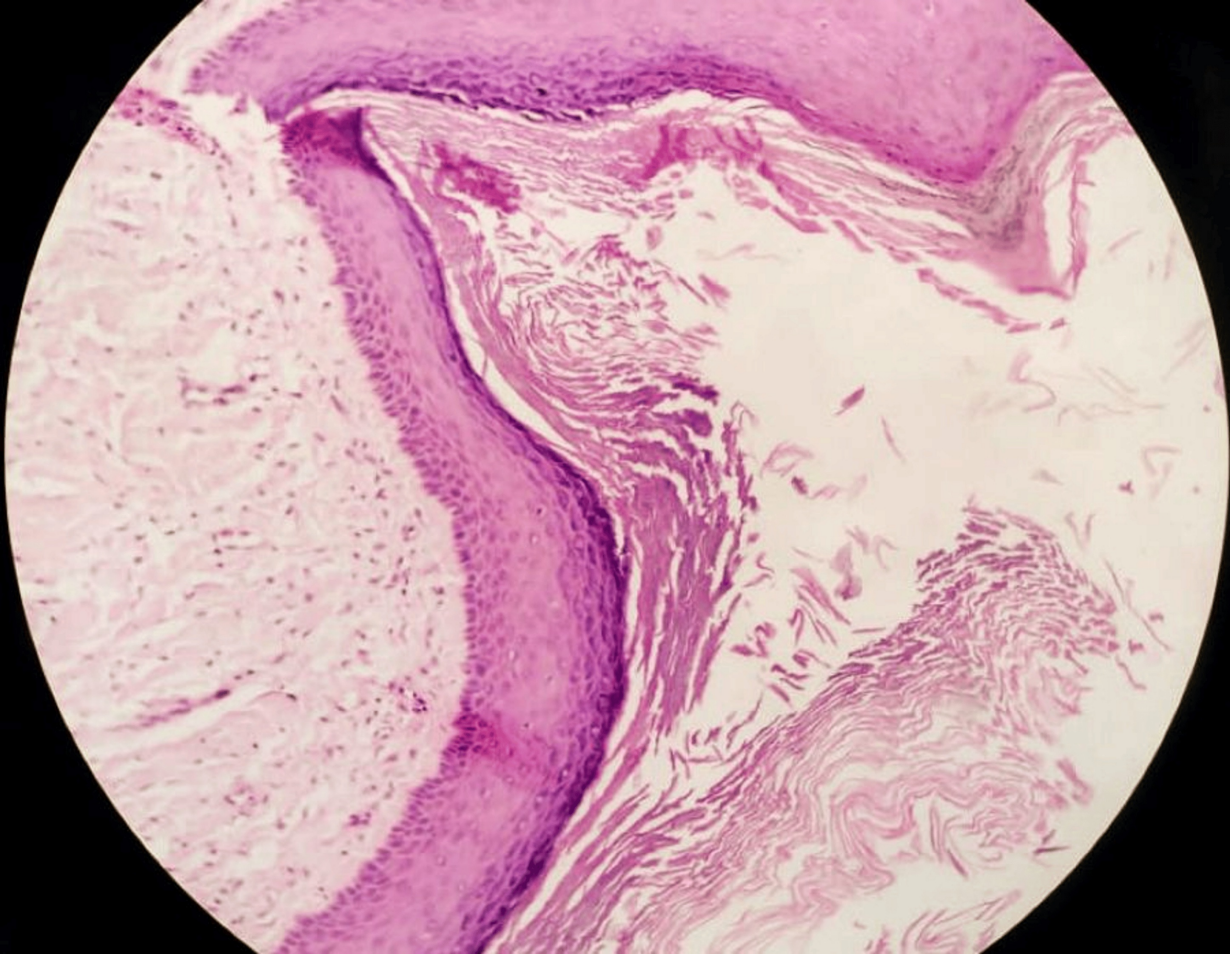
10X Hematoxylin & eosin staining Microscopic features showing a thin epithelial lining, permanent keratohyalin granules are present beneath the orthokeratotic surface, and flakes of orthokeratin are present in the lumen. The basal epithelial layer does not demonstrate palisading, which confirms the OOC and not OKC.

Microscopic examination revealed a cyst lined by orthokeratinized stratified squamous epithelium. A prominent granular cell layer was present, and the basal cell layer was flat to cuboidal without palisading. No satellite cysts, budding, or epithelial daughter cysts were seen (Figure 4). The findings suggested the diagnosis of an OOC.

Follow-up and outcome

The postoperative course was uneventful. The patient was followed up clinically and radiographically for 12 months with no evidence of recurrence. At the 12-month radiographic follow-up, the surgical site demonstrated progressive osseous healing characterized by substantial bone fill within the original defect, re-establishment of trabecular pattern, and early cortical plate remodeling. The overall defect size had noticeably decreased, indicating a favorable and stable healing trajectory.

## Discussion

An OOC is an uncommon cyst that shows distinct clinical and histopathologic characteristics compared to OKCs. The epithelium is orthokeratinized with a well-developed granular layer and lacks the corrugated surface and palisaded basal cells typical of OKCs [5].

Most OOCs are discovered incidentally, most often in young adults (2nd to 3rd decades), but can occur in adolescents and older adults. They are usually unilocular radiolucencies associated with impacted teeth [6]. Unlike OKCs, which have a high recurrence rate and potential for aggressive behavior, OOCs are more indolent and exhibit minimal recurrence following simple enucleation [7].

Differentiation between OOCs and other odontogenic cysts, especially OKCs and dentigerous cysts, is crucial. While radiographically similar, definitive diagnosis relies on histopathological examination. Immunohistochemical markers such as Ki-67 and p53 are generally lower in OOCs than in OKCs, supporting their less proliferative and aggressive nature [7,8].

Multiple OKCs usually occur in nevoid basal cell carcinoma, but our case presents multiple OOCs, which is a rare case to be reported with OOCs in both the maxilla and mandible in a late adolescent female patient [9].

## Conclusions

An OOC is a distinct pathological entity that differs from the more aggressive OKC in both clinical behavior and histopathological presentation. Accurate diagnosis is vital, as it influences treatment strategy and prognosis. The OOC typically follows a benign course with low recurrence rates when managed through conservative surgical enucleation, which is effective in the majority of cases; however, treatment should be tailored to individual presentations, and long-term follow-up is recommended. Awareness of its unique features can help prevent overtreatment and misclassification as an OKC or another odontogenic lesion.

Histopathological examination remains the gold standard for diagnosis, emphasizing the need for biopsy and microscopic evaluation in suspected cystic lesions of the jaws. Long-term follow-up, although precautionary, is advisable given the limited recurrence potential. As more cases are documented, a clearer understanding of its biological behavior will aid in refining clinical protocols and management guidelines. This case emphasizes the importance of thorough clinico-radiographic evaluation and histopathological confirmation for accurate diagnosis and appropriate management.
